# Echocardiographic Predictors of All-Cause Mortality in Patients with Hypertrophic Cardiomyopathy following Pacemaker Implantation

**DOI:** 10.1155/2020/2923767

**Published:** 2020-02-14

**Authors:** Nixiao Zhang, Wei Hua, Xiaoping Li, Yiran Hu, Hongxia Niu, Chi Cai, Min Gu, Xuhua Chen, Shu Zhang

**Affiliations:** ^1^Cardiac Arrhythmia Center, Fuwai Hospital, National Center for Cardiovascular Disease, Chinese Academy of Medical Sciences and Peking Union Medical College, Beijing 100037, China; ^2^Hospital of the University of Electronic Science and Technology of China and Sichuan Provincial People's Hospital, Chengdu 610072, China

## Abstract

**Objectives:**

To examine the association between the echocardiographic parameters measured as left atrial diameter (LAD) and left ventricular end-diastolic diameter (LVEDD) and long-term risk of all-cause mortality in adults with hypertrophic cardiomyopathy (HCM) following pacemaker implantation.

**Methods:**

A total of 94 adult patients with HCM who underwent pacemaker implantation from November 2002 to June 2013 in our Arrhythmia Center for symptomatic bradycardia and did not receive an implantable cardiac defibrillator (ICD) or cardiac resynchronization therapy (CRT) during follow-up were retrospectively extracted.

**Results:**

After careful examination of the medical records, we retrospectively evaluated the clinical characteristics of 74 patients with LAD records (58.1 ± 14.9 years) and 76 patients with LVEDD records (57.6 ± 15.2 years). Based on the receiver-operating characteristic (ROC) curve, the values of LAD = 44 mm and LVEDD = 43 mm were identified to predict the all-cause mortality. In the Kaplan–Meier survival, LAD ≥44 mm and LVEDD ≥43 mm were both significantly associated with all-cause mortality (log-rank test *P* < 0.05). Cox regression analysis indicated that LAD ≥44 mm (HR 3.580; 95% CI = 1.055–12.148; *P*=0.041) was an independent predictor of all-cause mortality, while LVEDD ≥43 mm was not significantly associated with all-cause mortality. LVOTO was also significantly associated with all-cause mortality (HR = 0.166; 95% CI = 0.036–0.771; *P*=0.022).

**Conclusions:**

In HCM patients with pacemaker implantation, LAD ≥44 mm was an independent predictor of all-cause mortality.

## 1. Introduction

Permanent pacemaker implantation is an invasive method to treat the severe and/or symptomatic bradycardia, developing due to conditions such as sick sinus syndrome (SSS), high-degree atrioventricular block (AVB), and bradycardia in atrial fibrillation (AF), which is of great importance to symptom relief [1]. The long-term outcomes of this procedure seem to be controversial. Previous studies paid more attention to the number of intracardiac electrodes [2, 3], the mode of cardiac pacing (AAI, VVI, and DDD) [2], and the right ventricular pacemaker lead position and their survival differences [2, 4, 5]. Marchandise et al. [3] compared single-lead VDD and DDD pacing and found the overall survival after adjustment was not significantly different in the two groups. A study [2] from Germany showed that there was a gradual increase in the survival every decade (*P* < 0.0001), and differences in the pacing mode and the type of arrhythmia were not significant solely in the last decade during 30-year follow-up. Age, gender, and the symptoms leading to pacemaker implantation were identified as the independent prognostic factors of survival. However, the investigators did not do a subgroup analysis of patients with hypertrophic cardiomyopathy (HCM).

HCM is the most common genetic cardiomyopathy with a prevalence of 1/500 in general [6]. Patients with HCM may require pacemaker implantation for not only relieving the left ventricular outflow tract obstruction (LVOTO) but also improving the symptomatic bradycardia. Earlier studies on hypertrophic obstructive cardiomyopathy (HOCM) [7–11] have focused to a greater extent on the decrease in the LVOT gradient with dual-chamber permanent pacing and symptomatic improvement in patients refractory to medical treatment. Javidgonbadi et al. [9] found short AV delay pacing benefited most of the HOCM patients in the relief of symptoms and LVOTO, with a low risk of reintervention. However, AV sequential pacing is rarely used despite evidence from these studies. Few data on pacing implantation in the whole HCM population or patients with nonobstructive hypertrophic cardiomyopathy (NOHCM) were analyzed. Left atrial diameter (LAD) is positively related to heart failure (HF) development, atrial fibrillation (AF), coronary heart disease, stroke, and all-cause mortality in the general population [12–14]. In a study of patients with HCM, left ventricular end-diastolic diameter (LVEDD) was found to be significantly correlated with mortality [13]. However, the impact of LVEDD, as well as LAD, on the all-cause mortality has not been well evaluated yet in HCM patients after the pacemaker implantation. Therefore, we aimed to study the long-term survival implications of left-sided heart structures measured as LAD and LVEDD in HCM patients with a pacemaker.

## 2. Materials and Methods

Adult patients admitted to our Arrhythmia Center between November 2002 and June 2013 with the diagnosis of HCM and symptomatic bradycardia were enrolled. Eligible patients were those who underwent pacemaker implantation during hospitalization. Patients who failed the implantation or died in the hospital and who presented for the generator upgrading of an ICD or CRT throughout the follow-up period were excluded in this study. HCM was identified when the myocardial hypertrophy (maximum wall thickness ≥15 mm for the general population or ≥13 mm for those with a family history of HCM) was detected by image examinations in the absence of abnormal loading conditions [6, 15]. HOCM was defined by the left ventricular outflow tract gradient ≥30 mmHg at rest or after exertion [15, 16]. A pacemaker was placed in patients with HCM with symptomatic bradycardia attributable to sinus node dysfunction, atrioventricular block, or bradycardia in AF. We reviewed the electronic medical records of all the patients. The preoperative clinical characteristics were recorded.

A permanent pacemaker was implanted intravenously. The passive atrial lead was located at the right auricle, and the passive fixation ventricular lead was placed in the right ventricular apex, or the active fixation ventricular lead was screwed into the septum near the right ventricular outflow tract. On the first postoperative day, the atrioventricular (AV) interval and the output of pacemakers were well programmed, with a long AV interval to encourage more intrinsic AV conduction for patients with intact AV conduction and with an optimal AV interval to mimic a normal PR interval (150–200 ms) for patients with AV block and for patients with LVOTO.

The demographic characteristics (age and sex), affiliated diseases, echocardiography, vital signs (heart rate, systolic blood pressure, and diastolic blood pressure), and medication at baseline were all extracted. The affiliated diseases included hypertension, diabetes mellitus, coronary heart disease, atrial fibrillation, and AV block. The echocardiography parameters were represented as LVOTO, interventricular septum thickness, LAD, LVEDD, LVEF, pulmonary hypertension, and severe tricuspid regurgitation. LAD according to the guideline [17] was assessed by the parasternal long-axis view, using the anteroposterior linear dimension at end systole which was the standard for linear LA measurement in the prior clinical and research works [18–22]. Pulmonary hypertension was defined as the estimated systolic pulmonary artery pressure >40 mmHg [23]. Severe tricuspid regurgitation was defined according to the guideline [24].

Follow-up data were obtained from healthcare databases and phone calls. The last follow-up of survivors was in the year of 2018. Patients who underwent cardiac transplantation were considered to reach the endpoint. If a person was lost to follow-up, the date of the most recent clinical evaluation was recorded. The endpoint in this study was the combined all-cause mortality or cardiac transplantation.

Continuous variables are expressed as mean ± SD or medians (quartiles), and the comparison between the two groups was performed using Student's *t*-test and Mann–Whitney *U* test. Categorical variables were described as percentages. Pearson's *χ*^2^ was used to compare categorical variables between the two groups. Receiver-operating characteristic (ROC) curves were depicted to obtain the cutoff values of LAD and LVEDD. Therefore, the cutoff value of 44 mm in LAD and 43 mm in LVEDD were used. Kaplan–Meier survival curves were used to evaluate the survival time associated with the endpoint. Significance of the differences between the survival curves was based on the log-rank test. Cox regression analysis was used to identify the relationship between LAD, LVEDD, and all-cause mortality. Adjusted hazard ratio (HR), 95% confidence intervals (CIs), and *P* values were calculated. The baseline variables with a *P* value <0.1 were introduced into a multivariate Cox proportional hazards model. Statistical significance was established as *P* < 0.05. All the analyses were conducted using SPSS version 22.0 for Windows.

## 3. Results

A total of 94 adult patients with HCM and pacemaker implantation at baseline were included in this study. 74 patients with LAD records and 76 patients with LVEDD records were eligible. During follow-up (7.3 ± 3.4 years), 6 patients were lost and 18 patients reached the endpoint. The 24 patients all had the medical records of LAD and LVEDD. The baseline clinical and echocardiographic characteristics of the study population were listed according to the two categories of LAD and LVEDD (Table 1).

**Figure 1:**
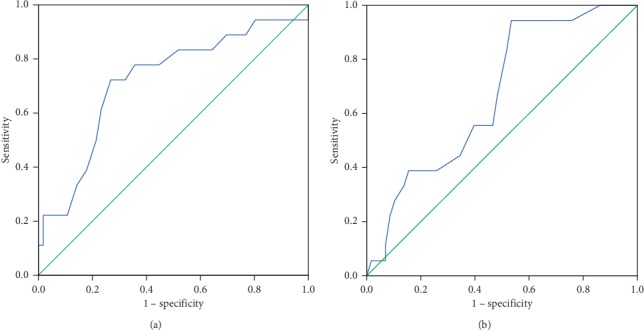
ROC curves with cutoff values of (a) 44 mm for LAD and (b) 43 mm for LVEDD.

A total of 74 patients with medical records of LAD were included. The mean age was 58.1 ± 14.9 years, with women accounting for 52.7%. The most prevalent comorbidity was AF, accounting for 41.9%, and then hypertension of 28.4%. Of these patients, 48.6% had LVOTO. The mean LAD and LVEDD were 41.7 ± 7.8 mm and 45.4 ± 6.5 mm, respectively. The majority underwent dual-chamber pacing (86.5%) and right ventricular (RV) apical pacing (98.6%). An LAD = 44 mm was identified to be a significant cutoff value through the ROC curve (Figure 1(a)). Compared to LAD <44 mm, LAD ≥44 mm was associated with a higher rate of atrial fibrillation (57.1% vs. 32.6%, *P*=0.038), a trend of lower LVOTO (35.7% vs. 56.5%, *P*=0.082), and a larger LVEDD (48.3 ± 6.5 mm vs. 43.7 ± 5.9 mm, *P*=0.003). The LVEF was lower with great significance in the LAD ≥44 mm group (59.9 ± 10.5% vs. 65.0 ± 7.0%, *P*=0.027). There were no significant differences in pulmonary hypertension and severe tricuspid regurgitation (*P* > 0.1). Patients with LAD ≥44 mm were prescribed more angiotensin-converting enzyme inhibitor (ACEI) or angiotensin-receptor blocker (ARB) medication (46.4% vs. 19.6%, *P*=0.014) and had a less tendency toward dual-chamber cardiac pacing (75.0% vs. 93.5%, *P*=0.057).

This group had two more patients with the medical records of LVEDD. The medical characteristics at baseline were similar to those based on LAD. An LVEDD of 43 mm was a significant cutoff value to predict all-cause mortality (Figure 1(b)). LVEDD ≥43 mm was significantly associated with a larger LAD (43.8 ± 8.4 mm vs. 38.3 ± 5.4 mm, *P*=0.003) and a lower LVEF (61.2 ± 8.9 mm vs. 66.1 ± 7.4 mm, *P*=0.016). Furthermore, patients with LVEDD ≥43 mm had a tendency of lower female distribution (43.8% vs. 64.3%, *P*=0.084) and *β*-blocker prescription (75.5% vs. 92.9%, *P*=0.053).

**Figure 2:**
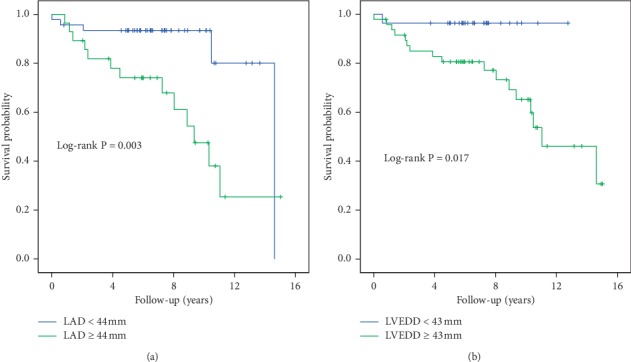
Kaplan–Meier estimates of the survival after pacemaker implantation (a) between HCM patients with LAD ≥44 mm and with LAD <44 mm and (b) between HCM patients with LVEDD ≥43 mm and with LVEDD <43 mm.

During follow-up, 13 patients (of whom one received cardiac transplantation) with LAD ≥44 mm and 5 with LAD <44 mm died, and 17 patients (of whom one underwent cardiac transplantation) with LVEDD ≥43 mm and 1 with LVEDD <43 died. In Kaplan–Meier analysis, cumulative hazard functions were significantly different between patients with LAD ≥44 mm and LAD <44 mm (log-rank test *χ*^2^ = 8.836; *P*=0.003) (Figure 2(a)). In parallel, patients with LVEDD ≥43 mm were associated with higher risks of all-cause mortality than those with LVEDD <43 mm (log-rank test *χ*^2^ = 6.661; *P*=0.017) (Figure 2(b)).

Multivariate Cox regression analysis revealed that LAD ≥44 mm was significantly associated with the increased risk of all-cause mortality (HR = 3.58; 95% CI = 1.055–12.148; *P*=0.041). In contrast, LVEDD ≥43 mm was not an independent predictor of all-cause mortality (HR = 4.141; 95% CI = 0.472–36.352; *P*=0.200). Furthermore, LVOTO was identified to be significantly related to the decreased risk of all-cause mortality (HR = 0.166; 95% CI = 0.036–0.771; *P*=0.022) (Table 2).

## 4. Discussion

In this study, we evaluated the all-cause mortality in HCM patients after pacemaker implantation and the baseline echocardiographic predictors measured as LAD and LVEDD. In the univariate analysis, the cutoff values of LAD = 44 mm and LVEDD = 43 mm were identified to predict the all-cause mortality. However, only LAD ≥44 mm was an independent predictor of all-cause mortality.

Interestingly, LVOTO as a confounding factor in Model 1 was found to be associated with the significantly decreased risk of all-cause mortality. The relationship between LVEF and all-cause mortality in Model 1 was not significant. The cause might be due to the influence of the factors that entered Model 1, such as ACEI/ARB administration and dual-chamber pacing. In previous studies, for HOCM patients, the pacing was of clinical and hemodynamic benefit, with the LVOT gradient significantly falling and the exercise tolerance and the symptoms of both dyspnea and angina being improved [8, 25]. Our study could not compare the echocardiographic parameters during follow-up with those at baseline because the echocardiographic parameters during follow-up were not available.

Sequential atrioventricular (AV) pacing, with the optimal AV interval to reduce the LVOT gradient or as a facilitated therapy in selected patients with LVOTO ≥50 mmHg requiring pacemaker implantation, is recommended in 2014 ESC guidelines of HCM [26]. Researchers in a study [9] of HOCM with long-term follow-up compared patients with short AV delay pacing to patients managed medically and treated with myectomy and found that the survivals were not different in the pacing and the conservative group, or in the myectomy group compared to the conservative group. Several studies have shown that patients with LVOT obstruction have a poorer prognosis in HCM patients [27, 28]. However, a study revealed that the differences in outcomes did not reach statistical significance in HCM patients with LVOTO compared to those without LVOTO [29]. In this study, we discovered that LVOTO was also significantly associated with all-cause mortality (HR = 0.166; 95% CI = 0.036–0.771; *P*=0.022), which may prompt that HCM patients with LVOTO had a better survival prognosis than those without LVOTO following the pacemaker implantation. To the best of our knowledge, there are at least 2 possible explanations for this difference. Right-sided heart excitation followed by left-sided heart excitation in patients with LVOTO markedly improves obstruction symptoms (such as palpitations, angina, and dyspnea [16]), hemodynamic abnormalities, and quality of life [7, 9, 10], which may neutralize the poor effects of RV apical pacing [30, 31]. Psychologically, more and more studies have focused on mental health and all-cause mortality [32–34]. In patients with LVOTO, the relief of symptoms was due to the improvement of the symptomatic bradycardia, as well as LVOTO, which might be more obvious than in those without LVOTO. Thus, they could get more comfort in spirit.

In HOCM patients, it has been reported that patients with reversed septal curvature (common in the young) benefited the least from the short AV delay pacing. Dimitrow et al. [35] enrolled 18 HOCM patients with a DDD pacemaker and found that the LVOT gradient reductions both at acute DDD pacing and at midterm follow-up (at least 6 months) were significantly greater in the patients with nonreversed septal curvature than those with reversed septal curvature. The latest article [36] published in the *Journal of the American College of Cardiology* reported that patients with the reversed curvature form were younger and less commonly had hypertension. In our study, the distributions of age and hypertension in different subgroups were similar. It has been unclear whether the reversed septal curvature is a risk factor for outcome events [36], let alone for the all-cause mortality. Another reason for no data on the reversed septal curvature in our study was due to the absence of uncommonly related echocardiography data. More prospective studies might be conducted to explore this relationship and the underlying mechanisms.

In terms of LAD, the prognostic value in predicting all-cause mortality in HCM remains controversial. Mild LA enlargement is commonly seen in HCM for the increased LV end-diastolic pressure. Both volume and pressure overload may lead to enlarged LA. Bostan et al. reported that LAD ≥41 mm predicted 13-year mortality with a sensitivity of 82% and specificity of 78%, respectively [13]. Nistri et al. [22] indicated LAD >48 mm was related to an HR of 1.9 for all-cause mortality in HCM patients (*P* < 0.05). However, Tani et al. [37] found LAD was not associated with the risk of cardiovascular events. Neither did Maron et al. [38] discover the association between LAD and cardiovascular mortality in patients with HCM. Few studies explored the cutoff values of LAD in predicting all-cause mortality in HCM patients with a pacemaker. This study indicated that an LAD of 44 mm was the most powerful predictor of all-cause mortality in HCM patients with a pacemaker.

LVEDD was largely affected by LV filling and contraction in HCM patients. Bostan et al. [13] identified LVEDD was significantly different between survivors and nonsurvivors in HCM patients (*P*=0.04). Nevertheless, LVEDD was not an independent predictor in the further analysis. Zhu et al. [39] enrolled 38 HOCM patients with previous alcohol septal ablation who underwent surgical septal myectomy and found postoperative LVEDD (HR = 1.14; 95% CI = 1.05–1.23; *P*=0.002) was an independent predictor of adverse events. Seiler et al. [40] indicated that HCM patients with chamber dilatation had a worse prognosis than those without, particularly for the quality of life. In this study, LVEDD ≥43 mm was not a predictor of all-cause mortality in HCM patients following pacing therapy after adjusting for the confounders at baseline.

There are several limitations in this study. Firstly, this was a retrospective study with some medical echocardiographic parameters missing, leading to fewer patients to be enrolled. Further prospective research would be needed to validate these results. Secondly, most of the patients (>98.5%) had RV apical pacing. Thus, it might be difficult to explore the relationship between the RV lead position and the all-cause mortality. Thirdly, we had no idea of the exact cumulative percent ventricular pacing and its influence on all-cause mortality, although the differences in AVB percentage between patients with LAD ≥44 mm and LAD <44 mm and between patients with LVEDD ≥43 mm and LVEDD <43 mm were not significantly different. Further studies need to keep an eye on the programming records during follow-up. Furthermore, because this was a retrospective study involving the medical records more than ten years old, the echocardiographic parameters during follow-up were lost. More prospective studies could identify the changes in LAD and LVEDD to predict the risk of all-cause mortality in these kinds of patients.

In patients with HCM following pacemaker implantation, LAD ≥44 mm was an independent predictor of all-cause mortality. LVOTO was significantly associated with the low risk of all-cause mortality. This might suggest that the management of LAD will benefit in all-cause mortality for these patients. It might be more helpful for patients with LVOTO to receive a permanent pacemaker. Further prospective studies with a larger sample size and cumulative percent ventricular pacing should be performed to evaluate these results.

## Figures and Tables

**Table 1:** Baseline characteristics of enrolled patients according to LAD and LVEDD.

Variables	All patients (*n* = 74)	LAD ≥44 mm (*n* = 28)	LAD <44 mm (*n* = 46)	*P* value	All patients (*n* = 76)	LVEDD ≥43 mm (*n* = 48)	LVEDD <43 mm (*n* = 28)	*P* value
Female, *n* (%)	39 (52.7)	14 (50.0)	25 (54.3)	0.716	39 (51.3)	21 (43.8)	18 (64.3)	0.084
Age, yrs	58.1 ± 14.9	60.8 ± 15.6	56.4 ± 14.4	0.223	57.6 ± 15.2	57.4 ± 14.9	58.0 ± 15.9	0.865
HTN, *n* (%)	21 (28.4)	7 (25.0)	14 (30.4)	0.615	21 (27.6)	16 (33.3)	5 (17.9)	0.146
DM, *n* (%)	11 (14.9)	4 (14.3)	7 (15.2)	0.913	11 (14.5)	9 (18.8)	2 (7.1)	0.294
CHD, *n* (%)	9 (12.2)	4 (14.3)	5 (10.9)	0.663	9 (11.8)	7 (14.6)	2 (7.1)	0.548
AF, *n* (%)	31 (41.9)	16 (57.1)	15 (32.6)	0.038	32 (42.1)	22 (45.8)	10 (35.7)	0.389
AVB, *n* (%)	17 (23.0)	5 (17.9)	12 (26.1)	0.414	18 (23.7)	11 (22.9)	7 (25.0)	0.837
LVOTO, *n* (%)	36 (48.6)	10 (35.7)	26 (56.5)	0.082	37 (48.7)	20 (41.7)	17 (60.7)	0.109
IVST (mm)	16.0 (13.0–21.0)	16.0 (12.3–19.0)	16.0 (14.0–21.0)	0.345	16.0 (13.0–21.0)	16.0 (12.0–20.0)	16.0 (14.0–24.0)	0.274
LAD (mm)	41.7 ± 7.8	—	—	—	41.7 ± 7.8	43.8 ± 8.4	38.3 ± 5.4	0.003
LVEDD (mm)	45.4 ± 6.5	48.3 ± 6.5	43.7 ± 5.9	0.003	45.7 ± 6.6	—	—	—
LVEF (%)	63.1 ± 8.8	59.9 ± 10.5	65.0 ± 7.0	0.027	63.0 ± 8.7	61.2 ± 8.9	66.1 ± 7.4	0.016
Pulmonary hypertension, *n* (%)	5 (6.8)	3 (10.7)	2 (4.3)	0.360	6 (7.9)	4 (8.3)	2 (7.1)	>0.999
Severe tricuspid regurgitation, *n* (%)	6 (8.1)	4 (14.3)	2 (4.3)	0.191	6 (7.9)	4 (8.3)	2 (7.1)	>0.999
SBP (mmHg)	120.0 (110.0–131.25)	120.0 (110.0–133.8)	120.0 (107.5–131.3)	0.866	120.0 (110.0–130.0)	120.0 (110.0–130.0)	120.0 (102.5–137.5)	0.732
DBP (mmHg)	74.5 (64.8–80.0)	70.0 (65.5–80.0)	80.0 (64.8–80.0)	0.458	74.5 (65.8–80.0)	70.0 (65.8–80.0)	78.0 (65.5–80.0)	0.455
HR (bpm)	64.5 (57.8–72.0)	64.5 (57.8–76.3)	64.5 (57.8–71.3)	0.793	64.0 (57.3–71.8)	63.5 (57.3–71.5)	68.0 (57.8–71.8)	0.351
*Medications*								
Β-blocker, *n* (%)	60 (81.1)	23 (82.1)	37 (80.4)	0.856	62 (81.6)	36 (75.0)	26 (92.9)	0.053
CCB, *n* (%)	35 (47.3)	10 (35.7)	25 (54.3)	0.119	36 (47.4)	21 (43.8)	15 (53.6)	0.408
ACEI/ARB, *n* (%)	22 (29.7)	13 (46.4)	9 (19.6)	0.014	22 (28.9)	17 (35.4)	5 (17.9)	0.103
AMIO, *n* (%)	10 (13.5)	5 (17.9)	5 (10.9)	0.616	10 (13.2)	6 (12.5)	4 (14.3)	0.999
Warfarin, *n* (%)	7 (9.5)	4 (14.3)	3 (6.5)	0.268	7 (9.2)	6 (12.5)	1 (3.6)	0.250
Dual-chamber pacing, *n* (%)	64 (86.5)	21 (75.0)	43 (93.5)	0.057	65 (85.5)	39 (81.3)	26 (92.9)	0.294
RV apical pacing, *n* (%)	73 (98.6)	28 (100.0)	45 (97.8)	>0.999	75 (98.7)	48 (100.0)	27 (96.4)	0.368

Data are presented as mean ± SD, median (interquartiles), or *n* (%). LAD, left atrial diameter; LVEDD, left ventricular end-diastolic diameter; HTN, hypertension; DM, diabetes mellitus; CHD, coronary heart disease; AF, atrial fibrillation; AVB, atrioventricular block; LVOTO, left ventricular outflow tract obstruction; IVST, interventricular septum thickness; LVEF, left ventricular ejection fraction; SBP, systolic blood pressure; DBP, diastolic blood pressure; HR, heart rate; CCB, calcium-channel blocker; ACEI/ARB, angiotensin-converting enzyme inhibitor/angiotensin-receptor blocker; AMIO, amiodarone; RV, right ventricular.

**Table 2:** Multivariate Cox regression analysis in predicting all-cause mortality.

Variables	HR	95% CI	*P* value
*Model 1: all patients with LAD records (n* *=* *74)*			
LAD ≥44 mm	3.580	1.055–12.148	0.041
AF	0.502	0.151–1.674	0.262
LVOTO	0.166	0.036–0.771	0.022
LVEDD (mm)	1.032	0.930–1.145	0.554
LVEF (%)	0.976	0.925–1.029	0.370
ACEI/ARB	0.487	0.113–2.097	0.334
Dual-chamber pacing	1.034	0.248–4.308	0.963
*Model 2: all patients with LVEDD records (n* *=* *76)*			
LVEDD ≥43 mm	4.141	0.472–36.352	0.200
Female	0.640	0.218–1.977	0.416
LAD (mm)	1.068	1.005–1.136	0.033
LVEF (%)	0.947	0.907–0.989	0.013
Β-blockers	2.666	0.688–10.337	0.156

HR, hazard ratio; CI, confidence interval; LAD, left atrial diameter; AF, atrial fibrillation; LVOTO, left ventricular outflow tract obstruction; LVEDD, left ventricular end-diastolic diameter; LVEF, left ventricular ejection fraction; ACEI/ARB, angiotensin-converting enzyme inhibitor/angiotensin-receptor blocker.

## Data Availability

The data used to support the findings of this study are available from the first author upon request.
